# Dysidenin from the Marine Sponge *Citronia* sp. Affects the Motility and Morphology of *Haemonchus contortus* Larvae In Vitro

**DOI:** 10.3390/md19120698

**Published:** 2021-12-09

**Authors:** Kelsey S. Ramage, Aya C. Taki, Kah Yean Lum, Sasha Hayes, Joseph J. Byrne, Tao Wang, Andreas Hofmann, Merrick G. Ekins, Jonathan M. White, Abdul Jabbar, Rohan A. Davis, Robin B. Gasser

**Affiliations:** 1 Griffith Institute for Drug Discovery, School of Environment and Science, Griffith University, Brisbane, QLD 4111, Australia; kelsey.ramage@griffithuni.edu.au (K.S.R.); k.lum@griffith.edu.au (K.Y.L.); sasha.hayes2@griffithuni.edu.au (S.H.); merrick.ekins@qm.qld.gov.au (M.G.E.); 2 Department of Veterinary Biosciences, Faculty of Veterinary and Agricultural Sciences, Melbourne Veterinary School, The University of Melbourne, Parkville, VIC 3010, Australia; aya.taki@unimelb.edu.au (A.C.T.); byrnej1@unimelb.edu.au (J.J.B.); tao.wang1@unimelb.edu.au (T.W.); a.hofmann@structuralchemistry.org (A.H.); jabbara@unimelb.edu.au (A.J.); 3 Max Rubner-Institut, Federal Research Institute of Nutrition and Food, 95326 Kulmbach, Germany; 4 Queensland Museum, South Brisbane, QLD 4101, Australia; 5 School of Chemistry and Bio21 Institute, The University of Melbourne, Parkville, VIC 3010, Australia; whitejm@unimelb.edu.au

**Keywords:** *Haemonchus contortus*, parasitic nematode, nematocidal, marine natural products, NatureBank, biodiscovery, extract library, sponge, *Citronia*, dysidenin, dysideathiazole

## Abstract

High-throughput screening of the NatureBank marine extract library (*n* = 7616) using a phenotypic assay for the parasitic nematode *Haemonchus contortus* identified an active extract derived from the Australian marine sponge *Citronia* sp. Bioassay-guided fractionation of the CH_2_Cl_2_/MeOH extract from *Citronia* sp. resulted in the purification of two known hexachlorinated peptides, dysidenin (**1**) and dysideathiazole (**2**). Compound **1** inhibited the growth/development of *H. contortus* larvae and induced multiple phenotypic changes, including a lethal evisceration (*Evi*) phenotype and/or somatic cell and tissue destruction. This is the first report of anthelmintic activity for these rare and unique polychlorinated peptides.

## 1. Introduction

Parasitic nematodes cause significant disease in livestock and affect hundreds of millions of livestock animals, such as sheep, goats, cattle and deer worldwide [1,2]. In most parts of the world, *Haemonchus contortus* (order Strongylida) is one of the most impactful parasites of small ruminants due to its blood feeding activity and pathogenic effects, leading to anaemia, reduced wool production and sometimes death [1,2]. Like most other strongylid nematodes, *H. contortus* is transmitted orally via the ingestion of grass contaminated with infective third-stage larvae (L3s). Following ingestion by the ruminant host, L3s exsheath (to become xL3s) and then develop to dioecious fourth-stage larvae (L4s) and adults within the stomach (abomasum) [2]. While a relatively small number of anthelmintic (antihelminth) drugs and a recently developed vaccine (Barbervax) against *H. contortus* are currently in use, resistance to anthelmintics has been widely reported in this and related parasites, and the efficacy of the vaccine can vary [3,4]. The heavy reliance on anthelmintic agents in parasite control programs, despite growing resistance, means that there is an imperative to discover and develop novel anthelmintics.

Some of our previous anthelmintic discovery work has identified nematocidal/nematostatic candidates in natural product extracts derived from plants or marine invertebrates [5,6]. To expand our biodiscovery efforts, we recently screened a collection of extracts (*n* = 7616) derived from marine invertebrates isolated from Australian waters in an established high throughput screening (HTS) assay for in vitro activity against *Haemonchus contortus* larvae [7]. We identified 58 active extracts that markedly reduced larval motility, achieving an overall “hit rate” of ~0.8% [7]. Of these 58 extracts, 16 significantly inhibited larval development and/or induced abnormal larval phenotypes. The majority of active extracts (54) were from sponges. ^1^H NMR fingerprinting was employed to dereplicate hits and to prioritise 29 samples for future chemical investigations. Herein, we report the chemical and biological investigations of a singleton hit extract from a species of *Citronia* that was identified in our HTS effort. *Citronia* is a poorly studied genus of sponge, with only two natural product chemistry papers reported to date. In one study, dysinosin A was isolated from *Citronia astra*, and found to act as a potent inhibitor of the blood coagulation cascade factor VIIa and an inhibitor of the serine protease thrombin [8]; in a subsequent investigation, citronamides A and B were isolated from the same sponge species and shown to have antifungal activity [9]. This paper constitutes the first investigation of anthelmintic activity from this sponge genus.

## 2. Results and Discussion

### 2.1. Bioassay-Guided Fractionation of the Citronia Extract

In order to identify the marine natural product(s) responsible for the anthelmintic activity of the extract from *Citronia* sp., we initiated an extraction and bioassay-guided fractionation investigation. The freeze-dried, ground *Citronia* specimen was sequentially extracted with *n*-hexanes, CH_2_Cl_2_:MeOH (8:2) and MeOH. The highly lipophilic hexane-soluble extract was discarded, and all the CH_2_Cl_2_ and MeOH extracts were combined and fractionated by reversed-phase C_18_ HPLC (H_2_O/MeOH/0.1% TFA) (Figure S1) [10], which yielded 60 fractions that were then evaluated for anthelmintic activity in the established xL3 motility assay [11].

**Figure 1:**
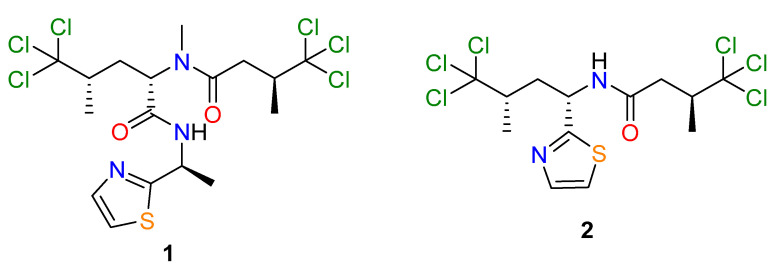
Chemical structures of dysidenin (**1**) and dysideathiazole (**2**).

**Figure 2:**
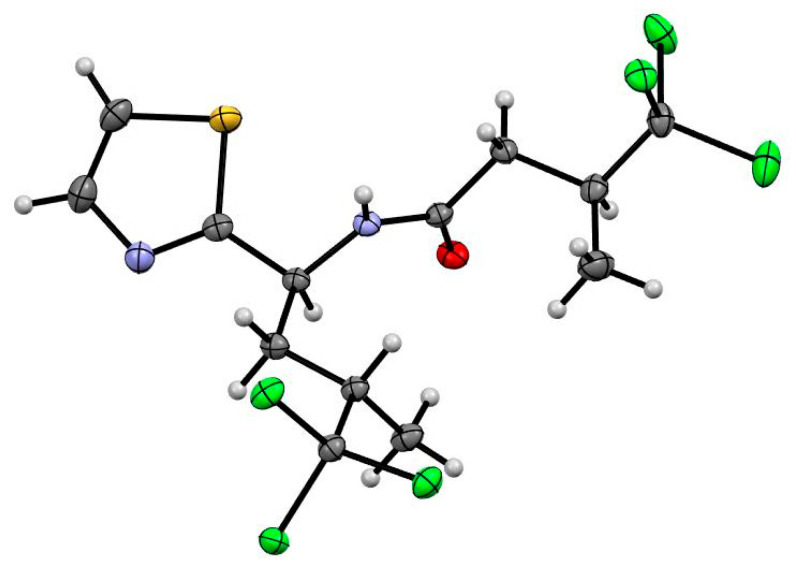
ORTEP drawing of dysideathiazole (**2**).

Fractions 50 to 55 and 57 each caused limited motility reduction (≤59%), but induced a curved (*Cur*) phenotype, with fraction 50 also inducing an eviscerated (*Evi*, lethal) phenotype in ~33.3% of larvae. C_18_ HPLC fractionation (H_2_O/MeOH/0.1% TFA) of fraction 50 yielded two major hexachlorinated compounds. Comparison of 1D NMR, UHPLC-MS (Figures S1–S8) and specific rotation data identified these compounds as the previously reported Dysideidae-derived metabolites, dysidenin (**1**) and dysideathiazole (**2**) (Figure 1) [12,13,14]; both compounds have had their absolute configurations determined, albeit via convoluted paths. For example, whilst the crystal structure of dysideathiazole (**2**) was first reported in the original isolation paper by Unson et al. [12], the data obtained did not allow unambiguous absolute configuration assignments to be made. However, chemical degradation of dysideathiazole and subsequent heavy atom derivatisation studies resulted in the absolute stereochemistry being determined for this molecule [12,13]. Fortuitously, during our isolation studies X-ray quality crystals of **2** were obtained. Subsequent low temperature data using Cu-Kα radiation out to higher resolution (2θ = 154.6°), gave a high-quality structure with a Flack parameter of 0.002(12) that enabled the absolute configuration of the natural product to be assigned (i.e., 2S, 5S and 7S); our data agreed favourably with the earlier assignment [12,14]. The thermal ellipsoid plot for dysideathiazole (**2**) is presented in Figure 2, which depicts 50% ellipsoids. Furthermore, from our studies it was noted that crystal packing for dysideathiazole is characterised by infinite chains of molecules related by a 2_1_ screw axis extending down the a-axis and held together by N-H…O hydrogen bonds and N…Cl halogen bonds (Figure S8).

### 2.2. Biological Evaluation of Dysidenin (***1***) and Dysideathiazole (***2***) Purified from Citronia

**Figure 3:**
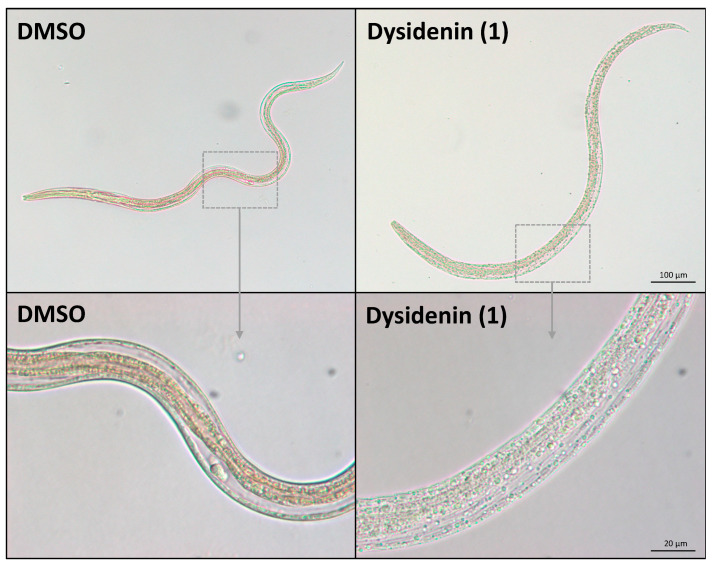
Photomicrographs of in vitro-raised fourth-stage larvae (L4s) of *Haemonchus contortus* treated with 100 μM of dysidenin (**1**) compared with a negative (DMSO-only) control, captured at 200-times (top) and 1000-times (bottom) magnification. Somatic (gut and muscle) cell destruction and tissue disintegration are visible (bottom right).

The two purified compounds, dysidenin (**1**) and dysideathiazole (**2**) (cf. Figure 1), were tested at a concentration of 100 µM for their anthelmintic effect on xL3s (in transition to L4s) and on in vitro-raised L4s. Although neither of the two compounds significantly reduced xL3 motility at 72 h, **1** inhibited larval development (from xL3 to L4) by 58% and induced abnormal phenotypes in 65% of worms (43% *Cur*, 14% *Evi* and 8% skinny (*Ski*)) at 168 h (Table 1). However, **2** did not induce developmental inhibition or phenotypic change, indicating that the active component within fraction 50 is dysidenin (**1**). When tested on in vitro-raised L4s, dysidenin (**1**) and dysideathiazole (**2**) reduced their motility by 61% and 25%, respectively, after 72 h of incubation (Table 1). Both compounds **1** and **2** induced a *Ski* phenotype in 50% and 28% of in vitro-raised L4s (Table 1), with IC_50_ values of 31 and 62 µM, respectively. This phenotype has previously been associated with damage of subcuticular musculature and mitochondria [15]. In addition, all L4s exposed to 100 µM of **1** exhibited marked destruction of somatic (gut and muscle) cells and tissue disintegration (Figure 3). Additional work is needed to evaluate the effect of these pure compounds on adult females and males of *H. contortus* isolated from, or in, an infected host animal (e.g., sheep).

This is the first report of anthelmintic activity of extracts from *Citronia*, and of dysidenin (**1**) and dysideathiazole (**2**) from any species other than *Dysidea herbacea* [12,13], although evidence of other biological activity has been reported for these polychlorinated peptides. Previous studies [16,17,18,19] demonstrated that dysidenin (**1**) and its C-5 epimer isodysidenin reduce iodide transport into thyroid tissues through an incomplete, reversible inhibition of mammalian sodium iodide symporters. Published findings [16,17] suggest that these two compounds may be pseudo-competitive for iodide in these symporters. Some moderate, but non-selective, inhibitory activity has been reported for dysidenin towards platelet-type 12-human lipoxygenase [20]. More recently, dysidenin (**1**) has been shown to inhibit bone morphogenetic protein-induced alkaline phosphatase in C2C12(R206H) mouse leg muscle cells with an IC_50_ value of 2.3 µM, but no toxicity to mammalian cells (up to 21.4 µM) has been observed [21]. While an LD_50_ of 5 mg/L (in water) was reported for the guppy fish (*Lebistes reticulatus*) [22] as evidence for ‘general’ toxicity, the methodology employed was not described by the authors, such that this claim warrants re-investigation.

The phenotypic changes induced here by dysidenin (**1**) in *H. contortus* larvae, particularly the lethal *Evi* phenotype in xL3s, and the somatic tissue disintegration and cell destruction in L4s, suggest that this compound has a unique mechanism of action, which requires future elucidation.

## 3. Materials and Methods

### 3.1. Chemistry Procedures

Melting points were measured using a Cole-Parmer apparatus (Antylia Scientific, Chicago, IL, USA) and were uncorrected. Specific rotations were recorded using a JASCO P-1020 polarimeter (Japan Spectroscopic Company, Tokyo, Japan). NMR spectra were recorded at 25 °C on a Bruker AVANCE III HD 800 MHz NMR spectrometer (Bruker, Billerica, MA, USA), equipped with a cryoprobe. The ^1^H and ^13^C chemical shifts were referenced to solvent peaks for CDCl_3_ (δ_H_ 7.26, δ_C_ 77.16). LRESIMS data were recorded on a Dionex Ultimate 3000 RS UHPLC (Thermo Fisher Scientific, Waltham, MA, USA) coupled to an ISQEC single quadruple ESI mass spectrometer (Thermo Fisher Scientific). Davisil C_18_-bonded silica (35–70 µm, 60 Å) were used for pre-adsorption prior to reversed-phase HPLC. The chromatography resin with pre-adsorbed material was packed into a stainless-steel guard Davisil cartridge (10 × 30 mm) and then attached to an HPLC column prior to fractionation. A Dionex Ultimate LC system was used for HPLC separations. Betasil C_18_-bonded silica (5 μm, 100 Å, 150 × 21.2 mm; Thermo Fisher Scientific) or Luna C_18_-bonded silica (5 µm, 90–110 Å, 250 × 10 mm; Phenomenex, Torrance, CA, USA) columns were used for reversed-phase HPLC separations. The ground sponge material was extracted at room temperature by continuous agitation using an orbital shaker (Bioline, Edwards Instrument Company, Narellan, NSW, Australia) set at 200 rpm. Solvents were removed from crude marine extracts with a Buchi R-144 rotary evaporator and from HPLC fractions using a GeneVac XL4 centrifugal evaporator. All solvents used for chromatography, mass spectrometry and polarimetry were HPLC grade and sourced from Honeywell Burdick & Jackson or Lab-Scan and were HPLC grade; H_2_O was filtered using an Arium^®^ Pro VF ultrapure water system (Sartorius, Göttingen, Germany).

### 3.2. Collection of Sponge Material

The sponge *Citronia* sp. was collected by SCUBA diving (1.8 m) from Ribbon Reef, Queensland, Australia, in November 2005. The sponge sample was immediately frozen at −20 °C upon collection and subsequently transported to the Griffith Institute for Drug Discovery, where the material was freeze-dried and ground into a fine powder and then stored in the NatureBank biota repository. A voucher specimen of *Citronia* sp. (QM G325135; operational taxonomic unit—OTU3186) has been deposited at the Queensland Museum, South Brisbane, Queensland, Australia.

### 3.3. Fractionation of the Citronia Extract

The freeze-dried and ground specimen of *Citronia* sp. (900 mg) was sequentially extracted with *n*-hexane (21 mL), CH_2_Cl_2_:MeOH (8:2, 21 mL) and MeOH (39 mL) at room temperature. The *n*-hexane extract was discarded, since it contained highly lipophilic (log *p* > 5) material, while all CH_2_Cl_2_ and MeOH extracts were combined and dried to give a crude extract (279 mg). Half of this extract was pre-adsorbed to C_18_-bonded silica (~1 g) and then packed into a guard cartridge for separation using a C_18_-bonded silica Betasil HPLC column. Isocratic solvent conditions of 90% H_2_O (0.1% trifluoroacetic acid, TFA)/10% MeOH (0.1% TFA) were employed for the first 10 min, followed by a linear gradient to 100% MeOH (0.1% TFA) over 40 min, and a final isocratic condition of 100% MeOH (0.1% TFA) for additional 10 min at a flow rate of 9 mL/min, collecting at 1 min intervals. In total, 60 fractions were collected.

From each of the 60 fractions, aliquots (2000 μge/μL) were transferred into a 384-well microtitre plate and tested in an established high throughput assay for *H. contortus* (see Section 3.6. Of the fractions that contained anthelmintic entities (nos. 50–55 and 57), the most abundant fraction (no. 50; 21 mg) was further purified by HPLC using a C_18_-bonded silica Luna column with isocratic solvent conditions of 90% H_2_O (0.1% TFA)/10% MeOH (0.1% TFA), employed for the initial 10 min, followed by a linear gradient to 100% MeOH (0.1% TFA) over 40 min, and, lastly, 100% MeOH (0.1% TFA) for additional 10 min at a flow rate of 4 mL/min; 1 min fractions were collected. This yielded the previously described marine natural products dysidenin (**1**, 3.0 mg, 0.33% dry wt, t_R_ = 35–36 min) and dysideathiazole (**2**, 2.0 mg, 0.22% dry wt, t_R_ = 33–34 min), in high purity (>95%).

Dysidenin (**1**): White amorphous solid; $\;{\left[\alpha \right]}_{D}^{24.3}$ −89.0° (*c* 0.365, CHCl_3_), lit. $\;{\left[\alpha \right]}_{D}$ −98° (*c* 0.5, CHCl_3_) [12], ^1^H and ^13^C NMR and UHPLC-MS data (see Figures S2–S4).

Dysideathiazole (**2**): Clear needles (25% EtOAc/75% *n*-hexane); *M* = 461.04 g/mol; mp 178–182 °C, lit. mp 176–177 °C [12], $\;{\left[\alpha \right]}_{D}^{24.3}$ −58.8° (*c* 0.17, CHCl_3_), lit. $\;{\left[\alpha \right]}_{D}$ −78.8° (*c* 2.07, CHCl_3_) [12]; ^1^H and ^13^C NMR and UHPLC-MS data (see Figures S5–S7).

### 3.4. X-ray Crystallography Analysis of Dysideathiazole

Intensity data for dysideathiazole (**2**) were collected using an Oxford Diffraction Synergy diffractometer with Cu-Kα radiation. An Oxford Cryosystems cooling device maintained the temperature at 100.0 K throughout the experiment. The structure was solved by direct methods and difference Fourier synthesis [23]. Hydrogen atoms were placed in their idealized positions and included in subsequent refinement cycles. Hydrogen atoms attached to heteroatoms were located from different Fourier maps and freely refined with isotropic displacement parameters. Thermal ellipsoid plots were generated in Mercury within the WINGX suite of programs [24,25]. The absolute configuration of dysideathiazole (**2**) was confirmed directly by the experiments detailed by Parson et al. [26].

Dysideathiazole (**2**): T = 100.0(10) K, λ = 1.54184 Å, Orthorhombic, space group P 2_1_2_1_2_1_ *a* = 9.4921(1), *b* = 10.4893(1), *c* = 19.7454(1) Å, *V* = 1965.96(2) Å^3^, *Z* = 4, *D_c_* = 1.558 Mg M^−3^ μ (Cu-K α) = 9.001mm^−1^, *F*(000) = 936, crystal size 0.31 × 0.19 × 0.13 mm^3^. θ_max_ = 77.30°, 68906 reflections measured, 4143 independent reflections (*R*_int_ = 0.0649) with *R*_final_ = 0.0246 [*I* > 2 σ(*I*), 4099 reflections] and *wR*(*F*^2^) = 0.0651 (all data), GOOF = 1.015. Absolute structure parameter: 0.002(12).

### 3.5. Preparation of Parasitic Nematode Larvae for Bioassays

The anthelmintic effects of fractions and purified compounds were tested on larvae of *H. contortus* (Haecon-5 strain). L3s were produced and stored using a well-defined protocol [11]—approved by the animal ethics committee of the University of Melbourne (permit no. 1714374). For use in the assay, L3s were exsheathed and sterilised by incubation in 0.15% (*v*/*v*) sodium hypochlorite (NaClO) at 38 °C for 20 min [27] and then washed five times in sterile saline by centrifugation at 500× *g* (5 min) at room temperature (22–24 °C). After the last wash, exsheathed L3s (i.e., xL3s) were suspended in Luria-Bertani broth (LB) containing 100 IU/mL of penicillin, 100 µg/mL of streptomycin and 0.25 µg/mL of amphotericin B (Fungizone; Thermo Fisher Scientific)—designated LB*. In vitro-raised L4s were produced by culturing xL3s for 168 h in LB* at 38 °C, 10% (*v*/*v*) CO_2_ and a relative humidity of >90%.

### 3.6. Bioassay for the Assessment of Anthelmintic Activity of Citronia Extract-Fractions

Individual fractions (*n* = 60) (Section 3.3) were tested for their anthelmintic effect on larvae (xL3s) of *H. contortus* using an established bioassay [11]. The assay was performed in triplicate. In brief, fractions in 40 μL of LB* (2000 μge/μL) were dispensed into the wells of sterile 368-well flat-bottom microtitre plates (cat. no. 3680; Corning, Corning, NY, USA) containing 80 xL3s; quadruplicate wells with no compound (LB* + 0.6% DMSO; negative control) or monepantel (Zolvix; Elanco, Greenfield, IN, USA), moxidectin (Cydectin; Virbac, Carros, France), monepantel/abamectin (Zolvix Plus; Elanco, Greenfield, IN, USA) and compound MIPS-0018666 (abbreviated here as M-666; ref. [28]) as positive-controls (20 µM). The motility of xL3s was measured at 90 h, and the development and phenotypic alterations of xL3s at 168 h. At 168 h, larvae in individual wells were fixed with 40 µL of 1% iodine and microscopically examined using a M80 light microscope (Leica, Wetzlar, Germany) at 60-times magnification to assess their development based on the presence or absence of a well-developed pharynx [27], as well as their morphology (phenotype) [7,11]. At 168 h, xL3s exposed to LB* with ≤0.6% DMSO are expected to reach the L4 stage in vitro within 168 h [10].

### 3.7. Bioassay for the Evaluation of Anthelmintic Activity of Purified Compounds

The compounds, dysidenin (**1**) and dysideathiazole (**2**) were individually tested for their anthelmintic effect on larvae (xL3s or in vitro-raised L4s) of *H. contortus* using an established bioassay [27]. Each assay was performed in triplicate on three different days. In brief, compounds were serially diluted in 50 μL of LB* (18-points, 2-fold dilution, 100 µM to 0.76 nM) and dispensed into the wells of sterile 96-well flat-bottom microtitre plates (cat. no. 3596; Corning) containing 300 xL3s or L4s; with six wells with no compound (LB* + 0.25% DMSO; negative control). A plate containing serial dilutions of monepantel (positive control) was prepared in the same manner. The motility of larvae was measured at 72 h, and the development and phenotypic alterations of xL3s at 168 h. At 168 h, larvae in individual wells were fixed with 25 µL of 1% iodine and microscopically examined using a DM1000 LED microscope (Leica, Wetzlar, Germany) at 100-times magnification to assess their development based on the presence or absence of a well-developed pharynx [27], as well as their morphology (phenotype) [7,11].

## Figures and Tables

**Table 1:** In vitro-activities of dysidenin (**1**) and dysideathiazole (**2**) against exsheathed third-stage larvae (xL3) and against in vitro-raised fourth-stage larvae (L4) of *Haemonchus contortus*. Maximum inhibitory values of each compound on the larval motility after 72 h incubation, the larval (xL3) development (in transition to L4) after 168 h and induced abnormal phenotypes at 72 h (in vitro-raised L4s) or 168 h (xL3s/L4s) are presented in reference to the values of control compound (monepantel) obtained under the same assay condition. Three independent experiments were conducted in all cases.

	xL3s			In Vitro-Raised L4s	
	Motility Inhibition (72 h)	Development Inhibition (168 h)	Abnormal Phenotype(s) Detected (168 h)	Motility Inhibition (72 h)	Abnormal Phenotype Detected (72 h)
Dysidenin (**1**)	nd	58%	*Cur* (43%), *Evi* (14%), *Ski* (8%)	61%	*Ski* (50%)
Dysideathiazole (**2**)	nd	nd	nd	25%	*Ski* (28%)
Monepantel	81%	100%	*Coi* (90%)	70%	*Ski* (78%)

nd, not detectable; *Cur*, curved; *Coi*, coiled; *Evi*, evisceration; or *Ski*, skinny (phenotypes).

## Data Availability

The data presented in this study are available in the article.

## Supplementary Materials

The following are available online at https://www.mdpi.com/article/10.3390/md19120698/s1, Figure S1: C18 HPLC chromatogram of the Citronia sp. extract with anthelmintic activity. Figure S2: 1H NMR (800 MHz) spectrum of dysidenin (1) in CDCl3. Figure S3: 13C NMR (200 MHz) spectrum of dysidenin (1) in CDCl3. Figure S4: UHPLC-MS data for dysidenin (1). Figure S5: 1H NMR (800 MHz) spectrum of dysideathiazole (2) in CDCl3. Figure S6: 13C NMR (200 MHz) spectrum of dysideathiazole (2) in CDCl3. Figure S7: UHPLC-MS data for dysideathiazole (2). Figure S8: Crystal packing drawing of dysideathiazole (**2**).
