# Supplementary material for: Dysidenin from the Marine Sponge Citronia sp. Affects the Motility and Morphology of Haemonchus contortus Larvae In Vitro
Source: Mar Drugs. 2021 Dec 9;19(12):698. doi: 10.3390/md19120698 (PMC8708643; doi:10.3390/md19120698)

Supplementary Materials

Dysidenin from the marine sponge *Citronia* sp. affects the motility and morphology of *Haemonchus contortus* larvae *in vitro*

Kelsey S. Ramage [1], Aya C. Taki [2], Kah Yean Lum [1], Sasha Hayes [1], Joseph J. Byrne [2], Tao Wang [2], Andreas Hofmann [2,3], Merrick G. Ekins [1,4], Jonathan M. White [5], Abdul Jabbar [2], Rohan A. Davis [1,*] and Robin B. Gasser [2,*]

[1] Griffith Institute for Drug Discovery, School of Environment and Science, Griffith University, Brisbane, QLD 4111, Australia; kelsey.ramage@griffithuni.edu.au (K.S.R.); k.lum@griffith.edu.au (K.Y.L.); sasha.hayes2@griffithuni.edu.au (S.H.); r.davis@griffith.edu.au (R.A.D.)

[2] Department of Veterinary Biosciences, Melbourne Veterinary School, Faculty of Veterinary and Agricultural Sciences, The University of Melbourne, Parkville, VIC 3010, Australia; aya.taki@unimelb.edu.au (A.C.T.); byrnej1@unimelb.edu.au (J.J.B.); tao.wang1@unimelb.edu.au (T.W.); a.hofmann@structuralchemistry.org (A.H.); jabbara@unimelb.edu.au (A.J.); robinbg@unimelb.edu.au (R.B.G.)

[3] Current address: Max Rubner-Institut, Federal Research Institute of Nutrition and Food, 95326 Kulmbach, Germany

[4] Queensland Museum, South Brisbane, QLD 4101, Australia; merrick.ekins@qm.qld.gov.au (M.G.E.).

[5] School of Chemistry and Bio21 Institute, The University of Melbourne, Parkville, VIC 3010, Australia; whitejm@unimelb.edu.au (J.M.W.).

* Correspondence: r.davis@griffith.edu.au (R.A.D.) and robinbg@unimelb.edu.au (R.B.G.)

Contents

S1 C_{18} HPLC Chromatogram of the *Citronia* sp. Extract with Anthelmintic Activity

S2 ^{1}H NMR (800 MHz) Spectrum of Dysidenin (**1**) in $CDCl_3$

S3 ^{13}C NMR (200 MHz) Spectrum of Dysidenin (**1**) in $CDCl_3$

S4 UHPLC-MS Data for Dysidenin (**1**)

S5 ^{1}H NMR (800 MHz) Spectrum of Dysideathiazole (**2**) in $CDCl_3$

S6 ^{13}C NMR (200 MHz) Spectrum of Dysideathiazole (**2**) in $CDCl_3$

S7 UHPLC-MS Data for Dysideathiazole (**2**)

S8 Crystal Packing Drawing of Dysideathiazole (**2**)

S1 C_{18} HPLC Chromatogram of the *Citronia* sp. Extract with Anthelmintic Activity

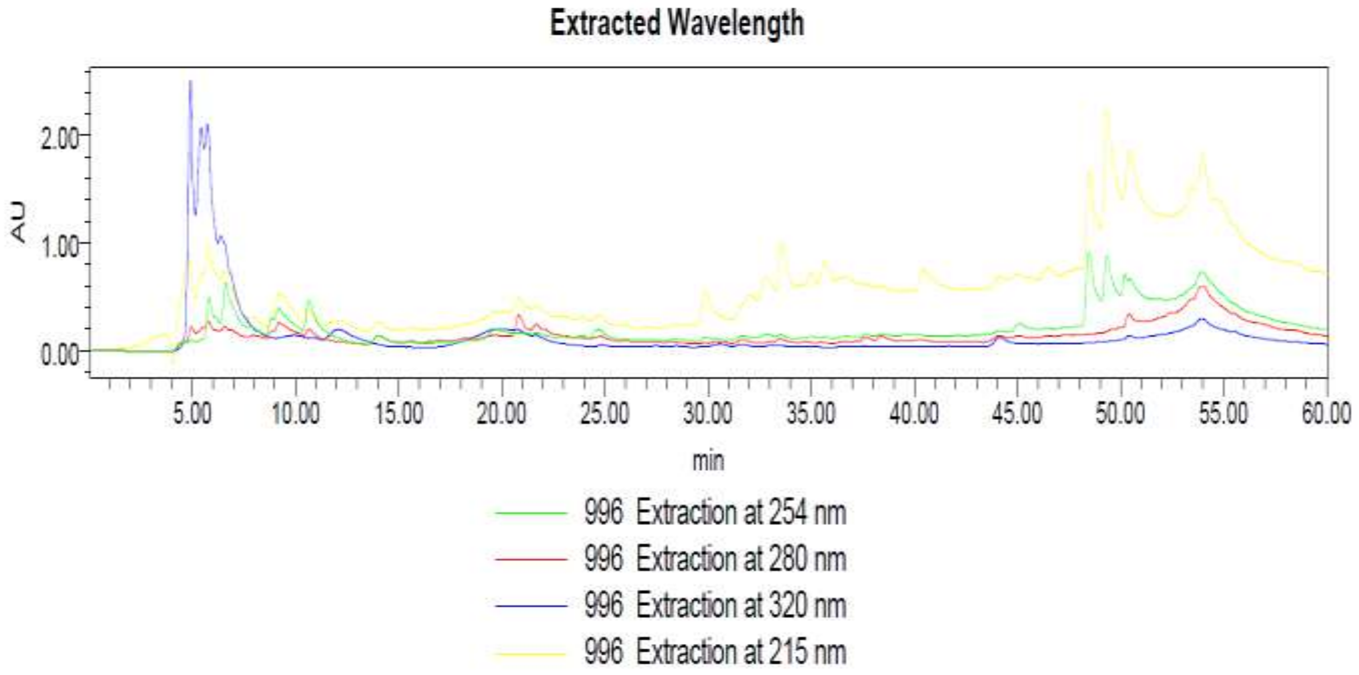

S2 ^{1}H NMR (800 MHz) Spectrum of Dysidenin (**1**) in $CDCl_3$

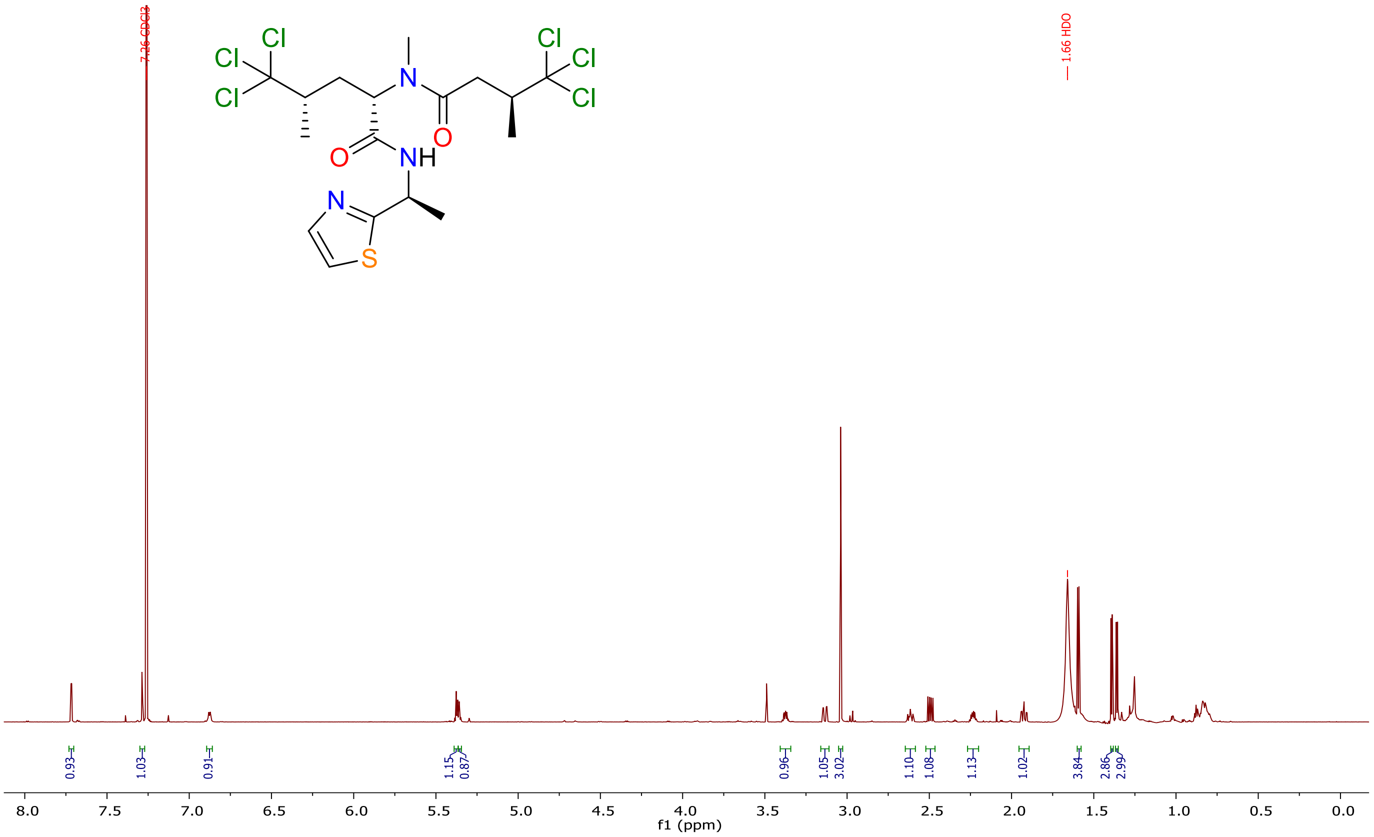

S3 ^{13}C NMR (200 MHz) Spectrum of Dysidenin (**1**) in $CDCl_3$

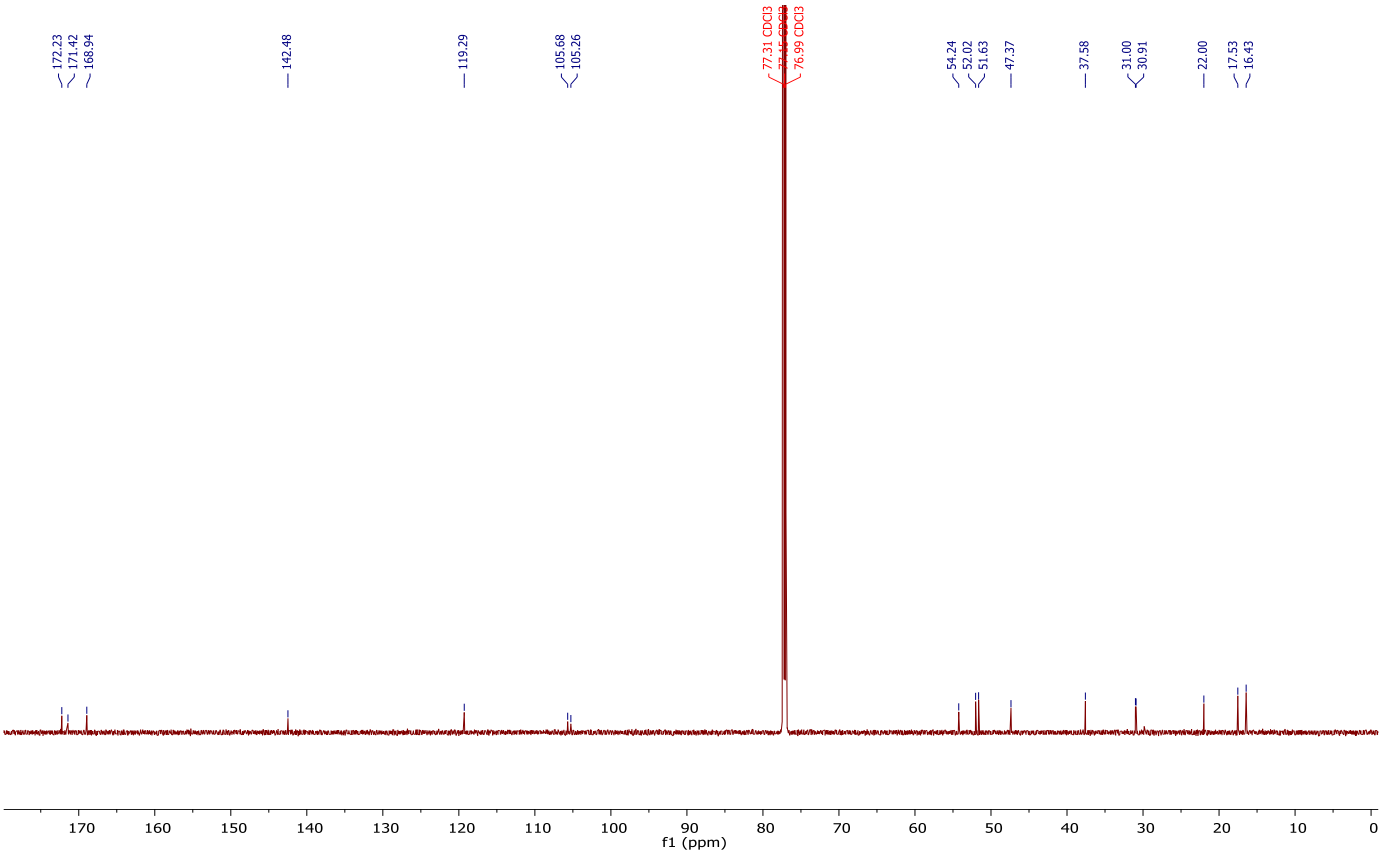

S4 UHPLC-MS Data for Dysidenin (**1**)

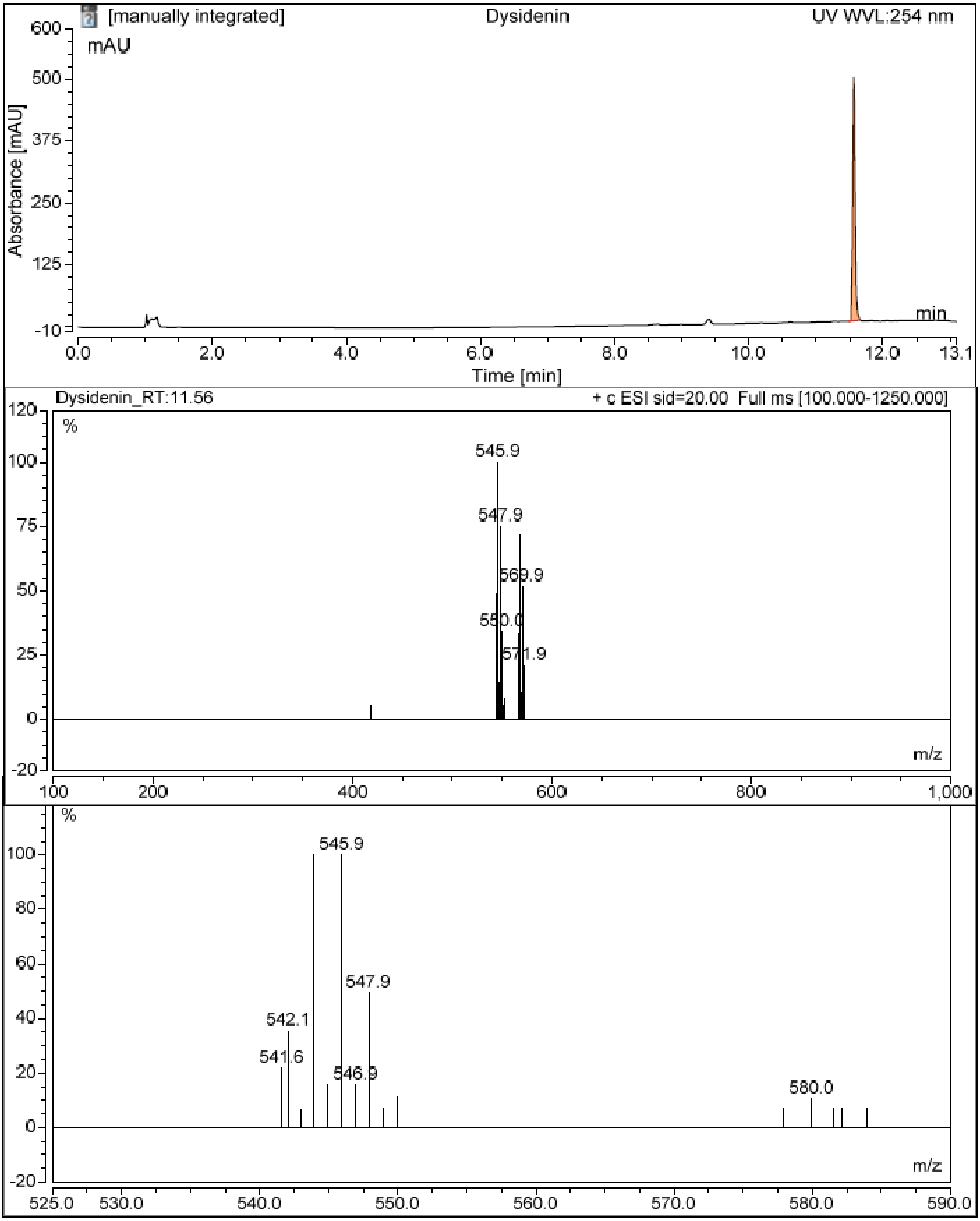

S5 ^{1}H NMR (800 MHz) Spectrum of Dysideathiazole (**2**) in $CDCl_3$

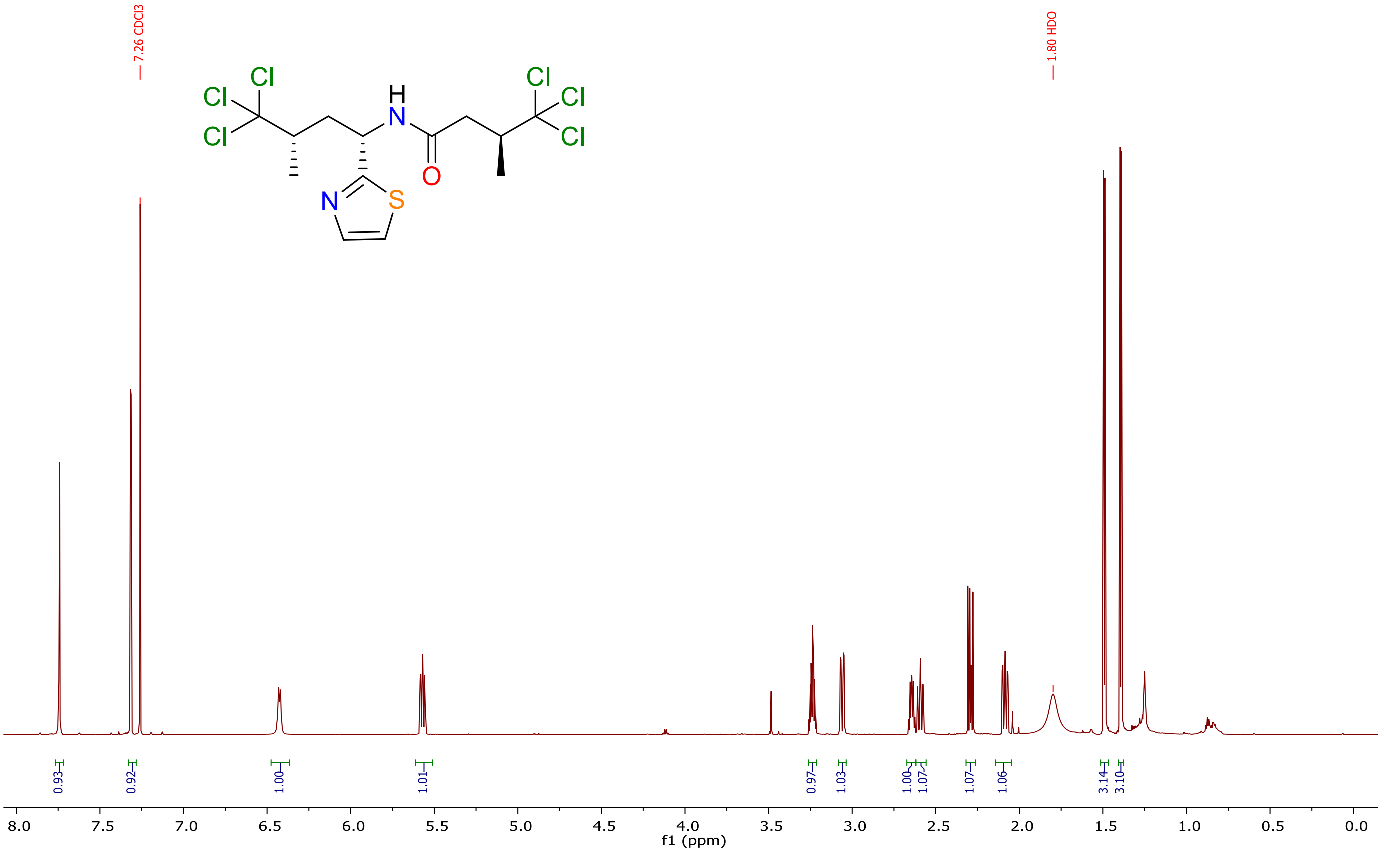

S6 ^{13}C NMR (200 MHz) Spectrum of Dysideathiazole (**2**) in $CDCl_3$

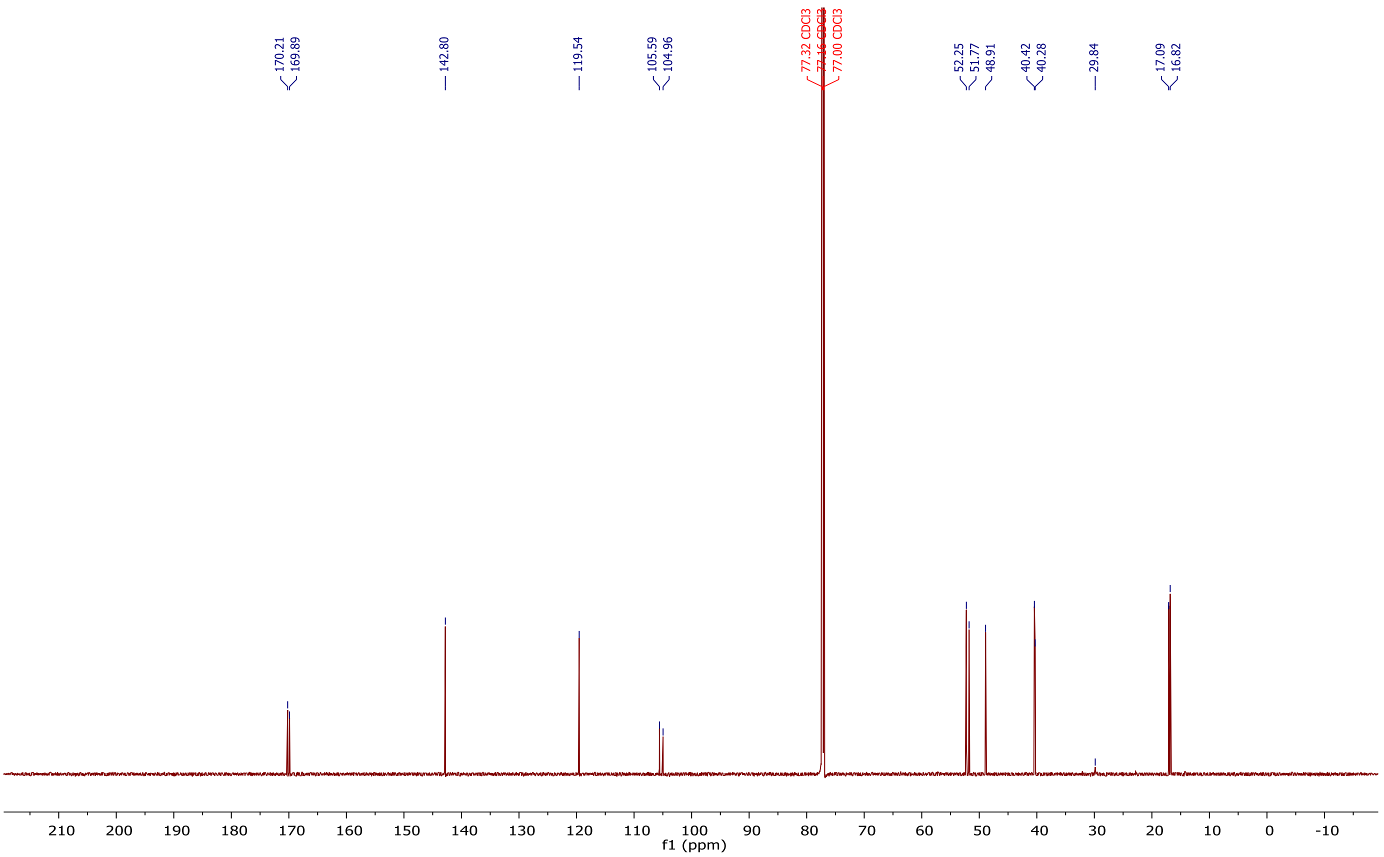

S7 UHPLC-MS Data for Dysideathiazole (**2**)

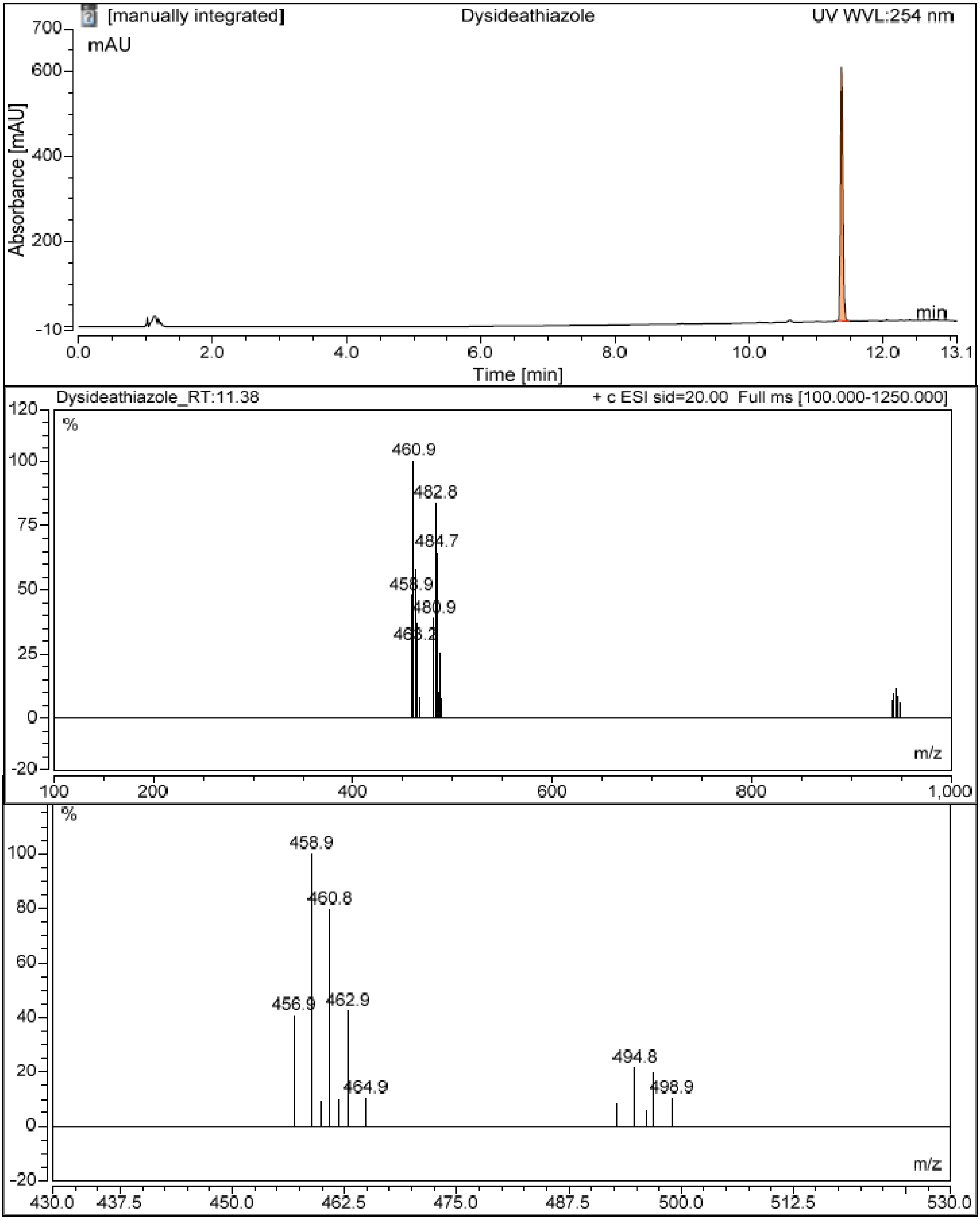

S8 Crystal Packing Drawing of Dysideathiazole (**2**)

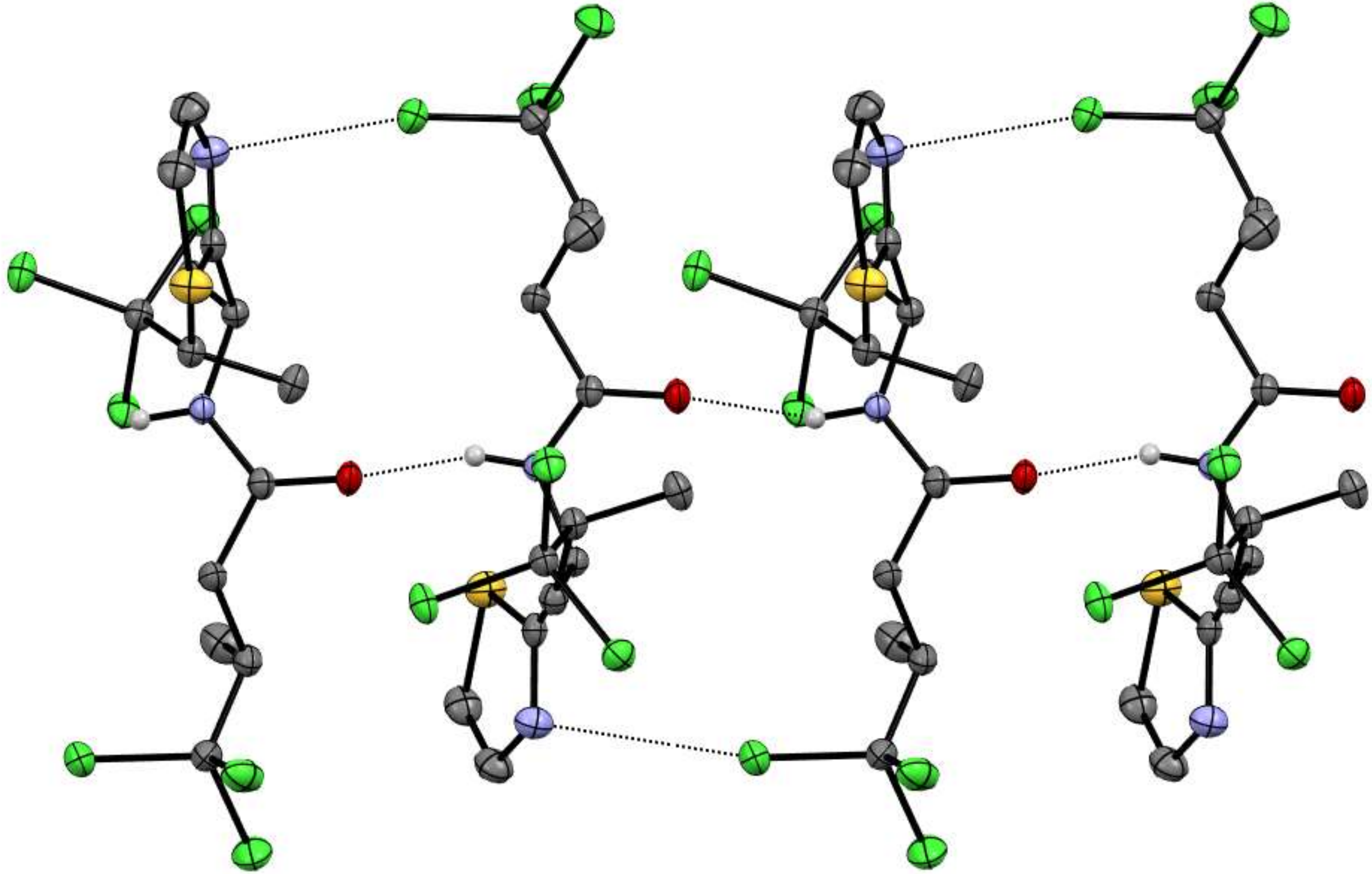

Supplement: Supplementary file 1 [file marinedrugs-19-00698-s001.zip › marinedrugs-1473300-supplementary.pdf]
